# Experiences of Older Adults Preparing for Their First Triathlon: “A Qualitative Study of the Participation in an Endurance Training Intervention.”

**DOI:** 10.1080/17482631.2021.1872824

**Published:** 2021-02-02

**Authors:** Bjørn Tore Johansen, Trine Brynjulfsen, Hilde Lohne Seiler, Monica Klungland Torstveit, Sveinung Berntsen Stølevik

**Affiliations:** Department of Sport Science and Physical Education, University of Agder, Kristiansand, Norway

**Keywords:** Running, cycling, swimming, elderly, well-being, being fit, sport participation, triathlon, personalized training

## Abstract

**Purpose**: The overall aim of the present study was to explore the experiences of older adult exercisers participating in an individualized training program lasting 3 months preparing for completing a triathlon competition.

**Methods:** Fourteen older Norwegian adults (median age (interquartile range, IQR) for males (N=10) and females (N=4) were 70.0 (65.0-75.5) and 57.5 (56.3-62.5) years, respectively) participated in 3-month individualized training program comprising three weekly sessions of running, cycling, and swimming. Both field- and laboratory-based testing were conducted. The participants attended two sports nutrition and competitive psychology seminars focusing on triathlon competition. The participants were interviewed in depth in three different focus groups. Thematic analysis was utilized to analyze the findings.

**Results:** Participants improved their performance in all field-based tests. After completion of the thematic data analysis the main finding and overarching theme of *well-being and being fit* emerged. Additionally, three main themes were identified: 1) *motivation*; 2) *progress and coping*; and 3) *breaking barriers*.

**Conclusion:** Psychological well-being and satisfaction of being fit seem to be vital to participate in a triathlon competition. Promoting specific age-appropriate participation in sports activities can be an effective strategy for promoting a healthy lifestyle among the elderly.

## Introduction

Sport is generally recognized as a beneficial leisure-time activity for children and youth (Eime et al., 2013). However, Jenkin et al. (2018) found that sports participation is also beneficial for social, physical, and intergenerational health of older adults. To encourage wider participation, better understanding of the barriers to and facilitators of participation in physical activity and sports for older adults is needed (Eime et al., 2016). The understanding of why older adults (people aged ≥50 years) participate in physical activity or sports is based mainly on research focusing on general recreational forms of leisure-time physical activity (Jenkin et al., 2018). Jenkin et al. (2018) emphasized that few studies have investigated barriers to and facilitators of older adults’ participation in sport, and most research is typically limited to studies of high-level competitive masters’ sport or in specific sports at the community level. Hence, there is a gap in the literature on population-based (e.g., elderly people) studies of sport participation and a lack of the perspective of the organizers of sport activities for older adults (Jenkin et al., 2018). To improve our understanding of older adults as consumers participating in sports, as well as sporting organizations as providers of sports, it is essential to identify the barriers to and facilitators of sports participation for older populations (Eime et al., 2016; Jenkin et al., 2018).

In Scandinavia, there is a general tendency for sections of the population (i.e., older adults) today to exercise and compete in sports including triathlon, cycling, and marathon (Overgaard et al., 2014). Reischer et al. (2016) claim that, for most active people today, endurance exercising is more about “completing” than “competing”. Consequently, it is of interest to understand how older adult exercisers experience participation in a training program that has been adjusted and aligned with the desire to complete a demanding endurance sport such as a triathlon competition.

Heo et al. (2013) suggested that involvement and competing in sports may have positive effects on physical competence, perceived physical ability, and instrumentality, which implies a tendency to face the world with self-determination and in an assertive setting. Such factors are expected to lead to the development of psychological well-being, which is a multidimensional concept that refers to having a positive image of self and life and includes aspects of self-esteem and life satisfaction (Leipert et al., 2011; Ryff, 2014). Well-being is a complex construct that concerns optimal experience and functioning in life (Kansky & Diener, 2017). Research on well-being is based on two general perspectives: the hedonic approach, which focuses on happiness and defines well-being in terms of pleasure attainment and pain avoidance and the eudaimonic approach (Ryff, 2014), which focuses on meaning and self-realization, and defines well-being in terms of the degree to which a person is fully functioning (Miquelon & Vallerand, 2006; Ryan & Deci, 2001). However, well-being is regarded a holistic concept (Bauer & McAdams, 2010; Huta, 2015) and hedonic and eudaimonic orientations play complementary roles and that both are important in contributing to overall psychological well-being (Huta, 2015).

In general, there is a lack of both quantitative and qualitative studies meaning that there is a major gap in the literature on the experiences of older adult exercisers participating in sport (Jenkin et al., 2018). Therefore, the overall aim of the present study was to explore the experiences of older adult exercisers participating in an individualized training program lasting 3 months preparing for completing a triathlon competition. In the present study, we have triangulated quantitative and qualitative methods to examine and describe their potential progression towards proficiency in the three triathlon sport activities. Using such a mixed-method research design (Creswell & Plano Clark, 2011) may contribute to build a more nuanced picture of the phenomenon studied.

## Materials and methods

### Participants and recruitment

The participants in the present study were contacted and recruited by email and/or personal contact by the research team. Twenty-five people aged >55 years (older adults; Jenkin et al., 2018) who had participated in an earlier research project were contacted originally. All potential participants were informed that they needed to provide written consent from their regular general practitioner (GP) confirming that their health status was sufficient to participate in a triathlon.

In total, 14 older Norwegian adults gave their consent to participate. Median age (interquartile range, IQR) for males (*N* = 10) and females (*N* = 4) were 70.0 (65.0–75.5) and 57.5 (56.3–62.5) years, respectively. All participants met the following inclusion criteria: aged >55 years; never previously competed in a triathlon competition; having sufficient health status to participate in the project; verified swimming ability; and a medical certificate from the GP for their participation in the project. The project plan for the intervention and interview guide for this study was approved by the Research Ethics Committee at the Faculty of Health and Sport Science at the University of Agder and Norwegian Centre for Research Data (59931).

### Intervention

All participants completed three training sessions per week for 3 months. In every training session of swimming, cycling, and running, the participants were divided into groups based on their skills and fitness level. Every training session was monitored by all participants using a heart-rate sensor, which was used to personalize the training intensity and duration. The entire intervention period prioritized providing individualized training by professional instructors and individual guidance. This procedure was chosen to minimize potential barriers and to facilitate the highest possible adherence by the participants.

To individualize the training program in terms of personal proficiency in and progression in the different triathlon sport activities, the participants completed two test periods before and after the training intervention. Testing included a laboratory test of cardiorespiratory fitness (maximal oxygen uptake) and a submaximal incremental test to identify the velocity at the second lactate threshold while running on a treadmill, which have been shown to correlate with endurance performance (Borg, 1970). In addition, participants completed three field tests including an outdoor 3000-m timed maximal running trial, 200-m timed swim trial in a pool, and an indoor 20-min maximal trial (mean power) on a cycle ergometer. Wilcoxon test was used to assess differences between pre- and post-test and the results will be presented in beginning of the Result section.

### Procedure

#### Focus group interviews

The participants were invited to participate in the interviews by email. All respondents contacted expressed an interest in taking part and were sent an information letter and were informed orally about the study and their rights. Written informed consent was obtained from all respondents.

Focus group interviews were chosen as a suitable way of fulfiling the aim of the study of physical fitness resources for preparing for and completing a triathlon competition because they often promote discussion and vigorous responses from participants (Stewart & Shamdasani, 2014). The participants were randomly allocated into three different groups, which aimed to take advantage of the group dynamics in an organized discussion in which the group could share their views of a specific experience or topic (Krueger & Casey, 2014). The focus group interviews were run as semi-structured interviews in appropriate settings two months after the participants had completed their triathlon competition. The participants knew each other well, and they received the overall topics to be discussed when they were asked to participate in the study. This procedure was meant to allow them to prepare for the focus groups and to assure them that would be no surprises when the interviews took place (Ericson et al., 2018). Each interview lasted between 45 and 70 min and resulted in a total of 130 pages of raw data (1.5-line spaced, Times New Roman 12-point font in Microsoft Office for Mac 2011).

### Instrumentation

#### Interview guide

A semi-structured interview guide was developed with the intention of exploring how the respondents experienced participating in the training program and completing the triathlon competition. The interview guide included four key themes. The questions were developed by the research team to elicit the respondents’ thoughts and experiences about: a) their reasons for participating in the project, b) their introduction to triathlon as a sport, c) their own exercise activity background, and d) potential lifestyle changes after the triathlon. Examples of interview questions were, “Can you tell us why you chose to join this program?” and “What have been the most positive or negative aspects of your participation in this project for people of your age?”

After the introductory phase of the focus group interviews, the participants were asked to talk freely about their experience of the following aspects: a) training sessions, b) training instructors, c) sports activities (i.e., swimming, cycling, and running), d) sports nutrition and competitive psychology seminars, e) completing a triathlon competition, and f) handling of potential conflicts as participants in the project.

To ensure that the responses were sufficiently in depth, the guidelines set out by Rubin and Rubin (1995), such as using various thematic categories to synthesize similar ideas and concepts related to the phenomenon studied, were followed and, subsequently, elaborating questions were asked to have the participants identify and describe the different dimensions and components of the study’s main research question. An example of these questions is, “Can you tell us more about the importance of mastery and progression in the training program to succeeding in completing the triathlon competition?”

### Data analysis

To ensure the validity and reliability of the process of analysing the data, the basic steps for reading verbatim transcripts put forward by Braun and Clarke (2006) were followed and included:

Familiarization with the data: The data were transcribed, read, and reread, and initial ideas were recorded.

Generating initial codes: Interesting features of the data were coded in a systematic fashion across the entire data set, and then the data were collated as relevant to each code.

Searching for themes: Codes were collated into potential themes, and all data relevant to each potential theme were collated.

Reviewing themes: The themes were checked to determine whether they worked in relation to the coded extracts (Level 1) and the entire data set (Level 2); a thematic “map” of the analysis was developed.

Defining and naming themes: Ongoing analysis was used to refine the specifics of each theme, and the overall story found in the analysis tells; clear definitions and names for each theme were identified.

Producing the report: This provided the final opportunity for analysis and included selection of vivid, compelling extract examples, final analysis of the selected extracts, relating back of the analysis to the research question and literature, and producing a scholarly report of the analysis.

Clarke and Braun (2015) emphasized the active role the researcher always plays in identifying patterns/themes, selecting which are of interest, and reporting them to the reader. In accordance with Clarke and Braun (2015) recommendation, we decided to analyse the data set based on the following three main themes that emerged from the analysis: 1) *motivation*, 2) *progress and coping*, and 3) *breaking barriers*. These fit within the study’s overarching theme as *well-being and being fit*. The extent to which a theme was considered “key” relates to whether it captures something important in relation to the overall research question (Braun & Clarke, 2006; Clarke & Braun, 2015). According to Tracy (2010), good qualitative research delves beneath the surface to explore and unearth issues that are assumed and implicit, and that are part of the average participant’s common sense.

**Figure 1.**
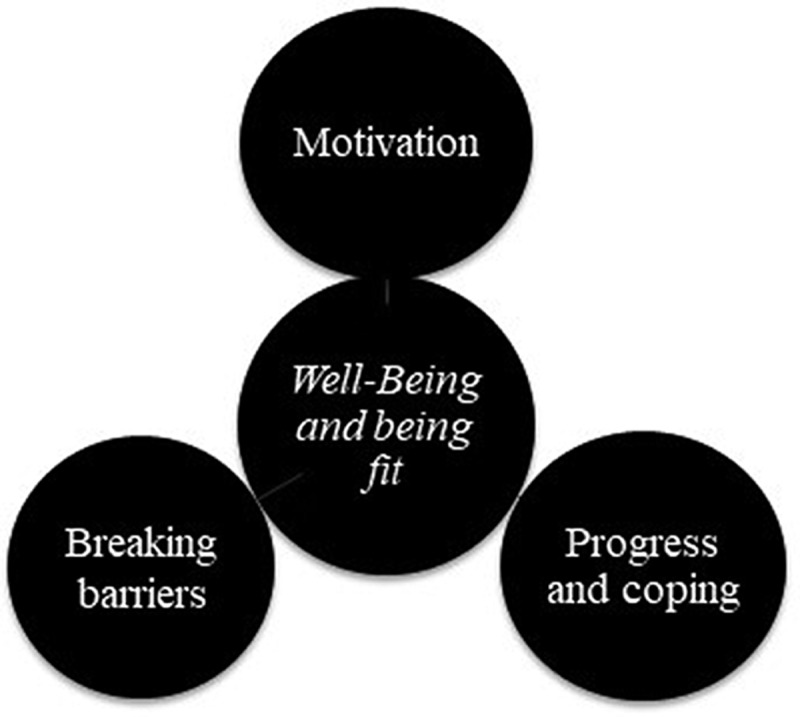
The three main themes that emerged from the analysis: 1) motivation, 2) progress and coping, and 3) breaking barriers coping. These fit within the study’s main finding and overarching theme well-being and being fit

The raw data consisting single phrases or statements were categorized, using NVIVO as qualitative analysis software, into subthemes related to the three main themes (Figure 1). The analysis was presented to the research team, and the members reached a collective agreement about main themes, subthemes, and data extracts. This process was repeated to gain a better overview to ensure data saturation (Braun & Clarke, 2019) and that the most exact meaning units and categories of descriptions were found (Malterud, 2012). The product of this process is the authors’ interpretation and perspectives of the respondents’ experiences. The recommended number of focus groups to gain data varies (Hennink et al., 2019), but should consist of four to twelve participants (Krueger & Casey, 2014). Our sample size of three focus groups and four to five participants in each of these groups ought to be sufficient for both code and meaning saturation (Hennink et al., 2019).

## Results

### Quantitative data

#### Intervention—objective assessments

Both field- and laboratory-based testing were conducted pre- and post-the 3-months individualized training intervention. Participants improved their physical fitness level in all the three field-based tests. More specifically, in the cycling trial, mean power significantly increased by 9.3% following training [pre(IQR): 167.0 (144.5–176.0) to post(IQR): 176.0 (167.0–193.0) (W), P < 0.005]. In both the running- and the swimming trial, time significantly decreased (−7.0% [pre(IQR): 20.6 (19.1–21.9) to post(IQR): 18.7 (17.9–21.0) (min), P < 0.002] and −13.9% [pre(IQR): 6.5 (5.7–8.1) to post(IQR): 5.9 (5.3–6.6) (min), P < 0.002], respectively). Whereas, in the laboratory-based submaximal incremental walking/running test, the velocity at the second lactate threshold did not significantly change (4.6%) [pre(IQR): 8.0 (7.3–8.4) to post(IQR): 8.5 (7.3–9.2) (km/h), P < 0.071].

### Qualitative data

#### Overarching theme—well-being and being fit

After completion of the thematic data analysis, three main themes emerged from the analysis: 1) *motivation*, 2) *coping and progress*, and 3) *breaking barriers*. These are suited within the study’s main finding and overarching theme *well-being and being fit* as suggested by the respondents (Figure 1):

#### From theme to subtheme

The data set from which the three main themes emerged was further coded according to the theoretical approaches used for forming the interview guide. This theoretical coding was used separately for all the three focus group interviews to identify possible subthemes within the data set for the different respondents. The process of reviewing themes started after the process of generating and searching for subthemes. This two-levelled process (Braun & Clarke, 2006) generated a thematic map of the analysis that was used for defining and naming subthemes across the data set obtained from the three focus groups. Despite the time-consuming process to note similarities and differences in experiences across all participants, it enabled us to identify the different subthemes within the different main themes.

#### Main theme: motivation and its subthemes

**Figure 2.**
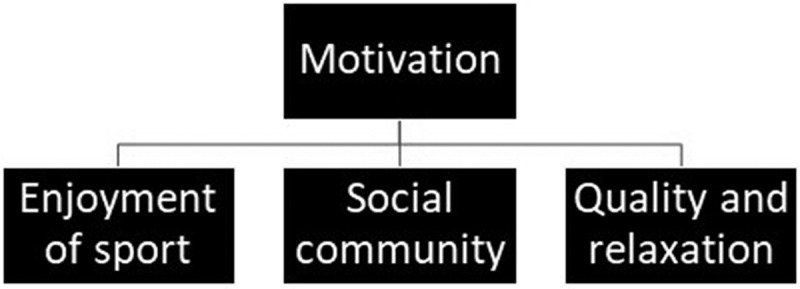
The main theme motivation with the three subthemes enjoyment of sport, social community, and quality and relaxation

From the in-depth analysis, motivation emerged as the main theme with three different subthemes: 1) enjoyment of sport, 2) social community, and 3) quality and relaxation. These subthemes were used to describe the respondents’ experiences within the overarching theme well-being and being fit (Figure 2).

The quotations listed below illustrate both the main theme and the associated subthemes. This simplified presentation of results of the thematic analysis are meant to clarify the ideas put forward in the figure. The following quotations from respondents illustrate the findings within subthemes and how these experiences are interwoven within the main theme.

### Enjoyment of sport

“Always loved to be physically active, never dreaded a training session or anything.”

“Exercise helps your mood.”

“Exercise has become a lifestyle.”

### Social community

“Always liked the context of exercising, the social setting of doing sports.”

“Probably did not have enough willpower to do this alone.”

“Nice social environment that supports you in triathlon training.”

### Quality and relaxation

“For the body, health and everything. For the psyche, not least.”

“Exercise has been my medicine for coping stress.”

“To me, it is all about well-being and being in good shape.”

#### Main theme: progress and coping and its subthemes

**Figure 3.**
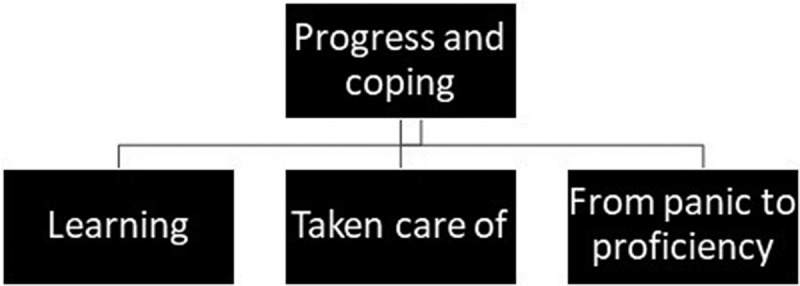
The main theme progress and coping with the three subthemes learning, taken care of, and from panic to proficiency

From the in-depth analysis, progress and coping emerged as the main theme with three subthemes: 1) learning, 2) taken care of, and 3) from panic to proficiency as descriptions of the respondents’ experiences within the overarching theme of well-being and being in good shape (Figure 3).

The following quotations from different respondents illustrate the subthemes and how these experiences are interwoven within the main theme:

### Learning

“Fun to have so much progress in all training sessions.”

“The project has helped me to be structured when planning training.”

“Happy to see the progress of others as well as my own.”

### Taken care of

“Nice with professional personnel that guide us through the activities, the follow-up, both in tests and in techniques.”

“Good swimming coach followed up each one pedagogically.”

“Assurance as participants because you get a full test of your body.”

### From panic to proficiency

“Had dreaded swimming and cycling but overhauled many competitors during both in the competition.”

“Was so afraid of the swimming in the sea, but now I am no longer afraid doing it so often I can.”

“Seemed to be too dangerous with cycling, but now I feel safe.”

#### Main theme: breaking barriers and its subthemes

**Figure 4.**
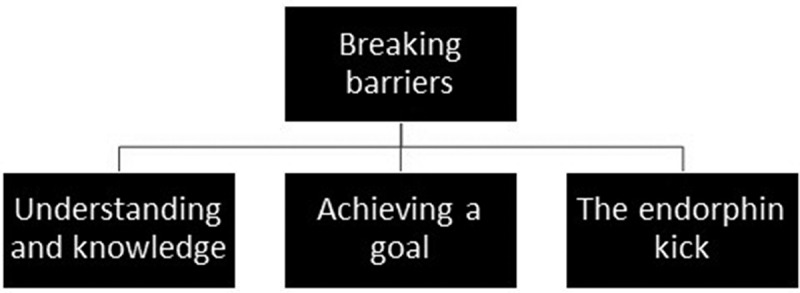
The main theme of breaking barriers with the three subthemes of understanding and knowledge, achieving a goal, and the endorphin kick

From the in-depth analysis, breaking barriers emerged as the main theme. Three subthemes emerged as description of the respondents’ experiences within the overarching theme well-being and being in good shape: 1) understanding and knowledge, 2) achieving a goal, and 3) the endorphin kick (Figure 4).

The following quotations from respondents illustrate the subthemes and how these experiences are interwoven within the main theme:

### Understanding and knowledge

“Nice with professional follow-up, both in tests and in techniques.”

“The project has helped me to be structured when planning training.”

“The competition-related training sessions were great; should have been more.”

### Achieving a goal

“I get now a good experience by variation in the training sessions.”

“Had great success in cycling, which I liked.”

“Was worried about swimming and cycling but passed many on both. Very satisfying.”

“Important for me to show that even if one turns 70–80 years, life is not over.”

### The endorphin kick

“20 kilometers on a bike is nothing anymore, I kind of get a special kick of it.”

“I have become addicted to endorphin intoxication after training sessions.”

“Exercise is a part of everyday life, gets bad unless I get trained.”

“Running has become a lifestyle.”

“Versatile exercise appeals to me instead of just walking the way I did before.”

## Discussion

The overall aim of the present study was to explore the experiences of older adult exercisers participating in an individualized training program lasting three months preparing for completing a triathlon competition. Further, we sought to examine and describe their potential progression towards proficiency in the three triathlon sport activities. In this study, well-being and being fit were two key elements in the respondents’ experiences of participating in the triathlon training program and being able to complete their first triathlon competition. In addition, the triathlon training intervention included an emphasis on individualized training, and personalized skill and technique development throughout the intervention period, including field- and laboratory-based testing before and after the training intervention. The results from the field- and laboratory-based testing indicated that the participants improved their physical fitness level significantly in the cycling-, running-, and swimming trial. However, in the submaximal incremental test, the velocity at the second lactate threshold did change but the change was not statistically significant. Nevertheless, taken together the findings of both the training intervention and the focus group interviews in the current study clearly suggest that the training program used succeeded in promoting the benefits such as psychological, social, and physical health derived from participating in triathlon activities.

Additionally, the training program with three weekly training sessions under the supervision of qualified personnel seems to have created an atmosphere for older adult exercisers to overcome classical barriers to sport participation, such as lack of appropriate playing opportunities, time constraints, and physical health and capabilities (Jenkin et al., 2018). Several of the respondents asked for more training sessions and given their progression and proficiency in the three different sports of swimming, cycling, and running, some participants wanted to undertake even more competition-related training sessions.

By progressing through and coping with the training sessions, and by breaking their individual barriers, the participants experienced benefits such as enjoyment, excitement, and relaxation. They also achieved an understanding of training and setting and meeting individual goals. Some even experienced the endorphin kick generated through participation in vigorous physical activity (Lechner, 2009; Leipert et al., 2011). Overall, the findings suggest that the personalized training approach allowed the participants to develop the psychological well-being and related feeling of being in good shape, which were crucial factors to their participation in the competition. In such situations, it is especially important to have goals and objectives, which gave their training sessions meaning, and to experience personal growth, manage everyday challenges, and find the strength to follow personal convictions (Ryff, 2014).

*Motivation* was one main theme that emerged from the analysis of the respondents’ experiences. Given the three subthemes of *enjoyment of sport, social community*, and *quality and relaxation*, it seems that the respondents were highly motivated to participate in the triathlon project. Although some of the participants perceived certain barriers, such as the lack of capability for swimming or cycling, they enjoyed, grew into, and adapted the training sessions by displaying a robust, intrinsic motivation (Deci & Ryan, 1985; Ryan & Deci, 2002).

There are reasons to believe that these participants fulfilled their three basic needs through autonomy, competence, and relatedness, and that the task climate experienced by the participants helped them to create a self-determined motivation (Ryan & Deci, 2006). The more practice people receive in developing and strengthening self-determination skills (Ryan & Deci, 2006), the more likely they are to make better decisions about training. Recent research on sport and physical activity participation has shown that the need for satisfaction is necessary for healthy development in older (56–75 years) people and their engagement, motivation, and well-being (Chan & Lee, 2019). The respondents’ experiences of the training program and training sessions indicated that the core dimensions of psychological well-being, such as self-acceptance, autonomy, personal growth, positive relationships, environmental proficiency, and purpose in life identified by Ryff (2014), seem to have been fulfilled by taking part in this triathlon project. The respondents’ report of happiness and well-being throughout the project period emphasized the positive motivational climate (Leipert et al., 2011; Ryan & Deci, 2001) achieved in this training group.

The main theme *progress and coping* with its subthemes *learning, taken care of*, and *from panic to proficiency* indicate that the respondents experienced psychological or mental well-being while training for and completing a triathlon competition. Mental well-being is largely accepted as covering two perspectives: the subjective experience of happiness and life satisfaction; and positive psychological functioning, good relationships with others, and self-realization (Tennant, Joseph, et al., 2007; Tennant, Joseph et al., 2007). Based on our findings, we suggest that both the hedonic and the eudaimonic perspective (Huta, 2015; Ryan & Deci, 2001) were present in the respondents’ experiences. The eudaimonic perspective includes the capacity for self-development, positive relationships with others, autonomy, self-acceptance, and competence (Ryan & Deci, 2001), and all these capacities were described clearly by the participants in the present study. Moreover, the respondents highlighted the importance of experiencing progress and coping during the training program, which indicated their ability to maintain a sense of autonomy, self-acceptance, personal growth, purpose in life, and self-esteem (see Ryff, 2014) while striving to meet their goals during training and completing the triathlon competition.

Additionally, by participating in this triathlon project, the participants developed a well-being platform that contributed to their understanding that staying mentally and physically healthy involves more than treating and preventing mental or physical illness. Indeed, the respondents’ lack of sport skills in swimming, cycling, and running before the triathlon project and the change in their perceptions about the necessary skills for mastering these different sports, clearly indicate the development of human potential that occurred during this intervention. This realization of their human potential may have contributed to the participants’ positive functioning in the training sessions, completing the competition, and life in general (Ryff, 2014). The development of human potential covers a wide range of cognitive aspects of mental health and the importance of being in good shape when participating in sport. Such intrapersonal benefits of participating in sports for older adults have recently been documented (e.g., Eime et al., 2016; Jenkin et al., 2018).

The third main theme *breaking barriers* with its subthemes *understanding and knowledge, achieving goal*, and *the endorphin kick* suggest that the participants reached a point of “no return” for participating in sport. Completing the training program and the triathlon competition seems to have led to the feelings of achievement and involvement in their athletic careers in these older adult exercisers. Consistent with results from previous studies suggesting that competition in athletic events provides both personal and social benefits for older adults (Heo et al., 2013; Siegenthaler & O’Dell, 2003; Smith & Storandt, 1997), the participants in present study reported various benefits (e.g., enjoyment, social interaction, self-actualization) associated with the sport participation. Several respondents reported that completing this triathlon project was their main purpose and overarching goal. In addition, after completing the triathlon competition, they reported that they had felt the endorphin kick which helped them to adjust their speed and intensity during the competition especially when passing other contestants along the triathlon course.

The participants’ knowledge about completing a serious endurance sport competition may have helped them to develop a unique ethos or subculture (Stebbins, 1997) as well as a distinctive social environment for sport participation. For some of the participants, their identity as senior athletes were important, and they could convey this to others by continuing to participate in local community running and cycling competitions. Several of the participants reported that, because of this triathlon project, they were currently competing in cycling and/or running. One respondent even attended the focus group interview wearing his sport attire before a competition. Moreover, previous research on runners has shown that the significance of their serious leisure identities (as long distance runners) increases after completion of an event because of different barriers such as pain and exhaustion are overcome (Shipway & Jones, 2007). The participants in the present study reported that their identity as older adult exercisers were sustained and they felt devoted to continuing training, participate in competitions, and further develop their skills in either swimming, cycling, or running. Furthermore, there are reasons to believe that mastering these triathlon activities might provide continuous mental and physical simulation to older adults (Wann et al., 2011) and, therefore, for the participants in the present study. The experiences of the respondents taking part in this triathlon project indicated that self-identifying as an older adult exerciser while transforming into a senior athlete may contribute to maintaining high cognitive and physical functions and, therein, creating an active lifestyle, which is one component of successful ageing (Heo et al., 2013).

### Strengths and limitations

All participants completed both the training program and the triathlon competition, and they were regularly involved in sport participation after the project. They should be considered subject matter experts who possess both an inside and outside perspective on the research context. The thematic style of the analysis used (Braun & Clarke, 2006; Clarke & Braun, 2015) may increase the validity and consistency of the data (Shenton, 2004). It could be argued that the exclusivity and homogeneous population itself is a strength. According to Malterud (2012), a study of six participants is enough to gain detailed descriptions of the phenomenon experienced by the participants. In our opinion, the empirical material succeeded in saturating (Braun & Clarke, 2019) the phenomenon examined, meaning that it was sufficient for revealing the main aspects of the experiences of these older adult exercisers participating in and completing the triathlon project. Denzin and Lincoln (2011) argued that a thick description and prolonged engagement are preconditions for establishing trustworthiness in a qualitative study. The strategic variation in the data generated from the 14 participants in the present study should be more than adequate to gain detailed descriptions of the phenomenon experienced by the respondents. Further, the researchers’ background and pre-understanding can be an advantage in qualitative research because of the access gained into the respondent’s everyday world (Kvale & Brinkmann, 2009).

This study has some limitations. The relatively limited number of participants in this study requires caution in the interpretation. All quotations used in this article were translated from Norwegian to English. To avoid possible limitations in the analysis because of language differences, the whole analysis process was completed in the original language (Van Nes et al., 2010). The focus group interviews were led by a researcher who was much younger than 50 years, which may have affected the responses given by the older adult participants and subsequent analysis (Ericson et al., 2018). The participants in the present study do not represent a diverse socioeconomic group, and a more heterogeneous population might provide further insights into the subcultural demands of participating in and completing a triathlon competition for senior exercisers of different ethnicity and socioeconomic status. Future research may wish to examine whether the findings presented here can be replicated in a sample in other senior athlete contexts such as in ball game sports. Accordingly, future research may benefit from a multifaceted data collection approach, which was not conceivable in the present study.

## Conclusion

This present study used a mix-method research design to examine the experiences of older adult exercisers who participated in a training program and completed a triathlon competition. The qualitative approach used allowed us to explore subjective reflections, and we would like to claim that our supplemented objective measurements combined with qualitative reconstructions add value to the scientific discussion on sport participation for older adult exercisers in a demanding endurance sport. Moreover, the focus group interviews with these participants provided us with a unique insight into the potential facilitators of participation by older adults in triathlon. Such facilitators were individualized training, personalized skill, and technique counselling, and field testing during the intervention period. Based on our results, we suggest that local sports clubs might take a stronger promotional approach in collaboration with senior and community centres to reach out to potential participants in triathlon sport activities. We recommend that sport policymakers increase the focus on older adults and that such opportunities can be developed further accordingly. In that way, the introduction and promotion of sports in general, and triathlon in particular, may provide a practical leisure-time physical activity option for older adults to help them maintain or improve their health, particularly their social health, which is important for people who are retired or living alone.

The results of our study suggest that psychological well-being and feeling of being in good physical shape are positively related to the depth of involvement in a personalized training program in older adults. Promoting specific age-appropriate opportunities for older people to participate in the sport of triathlon may be a new but effective strategy for encouraging a healthy lifestyle among elderly.
